# Prevalence and risk factors for vascular calcification based on the ankle-brachial index in the general population: a cross-sectional study

**DOI:** 10.1186/s12872-022-02668-9

**Published:** 2022-05-18

**Authors:** Shengnan Chen, Ning Li, Yajuan Gao, Hongli Jiang, Yan Shen

**Affiliations:** 1 Department of Blood Purification, Kidney Hospital, The First Affiliated Hospital of Xi’an Jiaotong University, West Yanta Road No. 277, Xi’an, 710061 Shannxi China; 2 Department of Ultrasound, The First Affiliated Hospital of Xi’an Jiaotong University, Xi’an, 710061 Shaanxi China; 3 Department of Nephrology, Kidney Hospital, The First Affiliated Hospital of Xi’an Jiaotong University, West Yanta Road No.277, Xi’an, 710061 Shannxi China

**Keywords:** Vascular calcification, Ankle-brachial index, General population, Risk factors

## Abstract

**Background:**

To investigate the prevalence of vascular calcification based on the ankle‐brachial index (ABI) value and analyse the risk factors for vascular calcification in the general population.

**Methods:**

A cross-sectional study was conducted to collect clinical, laboratory, and lifestyle data in individuals aged 30–70 recruited from the physical examination centre. The automatic arteriosclerosis detector was used to measure the ABI. Difference tests, correlation analyses, and multivariate logistic regression analyses were performed to identify risk factors for vascular calcification.

**Results:**

The overall prevalence of vascular calcification was 24.39% in 1033 subjects. The prevalence of vascular calcification in males was much higher than that in females (27.80% *vs.* 17.49%, *P* < 0.001). The differences in age, body mass index (BMI), systolic blood pressure (SBP), diastolic blood pressure (DBP), triglyceride (TG), high-density lipoprotein cholesterol (HDL-C), hypertension, and fatty liver disease were statistically significant in males (*P* < 0.05). The differences between serum uric acid (UA), total cholesterol (TC), TG, low-density lipoprotein cholesterol (LDL-C), estimated glomerular filtration rate (eGFR), alcohol consumption, exercise, and postmenopausal status were statistically significant in females (*P* < 0.05). Increased age (odds ratio (OR) = 1.028, 95% confidence interval (CI) 1.008–1.049, *P* = 0.007), increased BMI (OR = 1.238, 95% CI 1.147–1.337, *P* < 0.001) and elevated DBP (OR = 2.563, 95% CI 1.262–5.205, *P* = 0.009) were independent risk factors for vascular calcification in males after adjusting for confounding factors. Increased BMI (OR = 1.159, 95% CI 1.029–1.304, *P* = 0.015), elevated UA (OR = 1.545, 95% CI 1.077–2.216, *P* = 0.018), elevated LDL-C (OR = 1.044, 95% CI 1.060–1.027, *P* < 0.001), and a lack of exercise (OR = 2.402, 95% CI 1.073–5.373, *P* = 0.033) were independent risk factors for vascular calcification in females.

**Conclusions:**

The prevalence of vascular calcification based on the ABI value is also high in the general population of our centre. Increased age, BMI, and elevated DBP are independent risk factors for vascular calcification in males. Increased BMI, UA, LDL-C, and a lack of exercise are independent risk factors for vascular calcification in females. Attention should be given to strengthening the prevention and control of vascular calcification in the general population.

## Background

Cardiovascular disease (CVD) remains a significant cause of death globally [1]. With the ongoing epidemic of hypertension, diabetes, and chronic kidney disease (CKD) [2], the prevalence of CVD is still increasing every year [3], imposing a heavy socioeconomic burden and threatening human life all over the world. Arteriosclerosis, including atherosclerosis and vascular calcification, are common pathophysiological changes found in patients with CVD [3]. Both atherosclerosis and vascular calcification are well-known risk factors for cardiovascular events [4] and have a close association with the mortality rate of CVD patients.

The ankle-brachial index (ABI) is an important noninvasive method to evaluate vascular wall structure and functional changes [5] with several advantages including accuracy, cost-effectiveness, convenience, and requiring no radiation. The normal value of the ABI is considered to be 0.9–1.3 [6], and the clinical significance of ABI is primarily based on its ability to identify lower extremity ischaemic disease [7]. An ABI of less than 0.9 has been shown to have a high sensitivity and specificity for the diagnosis of peripheral artery disease (PAD) [8]. Furthermore, even in the absence of PAD symptoms, the ABI is also a prognostic marker for cardiovascular events and functional impairment [9]. Despite various studies over the past decades devoted to understanding the origin, diagnosis, and prevention of aortic calcification, there is a glaring lack of studies on systemic vascular calcification. However, arteriosclerosis caused by vascular calcification in the lower limbs occurs significantly earlier than that in the upper limbs [10]. Thus, a high ABI is a reliable indicator of the presence of early vascular calcification and is closely related to the increased risk, adverse outcomes, and all-cause mortality of CVD events [11]. Many studies have noted that an ABI of greater than 1.3 indicates the presence of a major form of arteriosclerosis, usually referred to as vascular calcification [9, 12–16]. Therefore, attention should be given to the evaluation of vascular status and related risk factors in populations with high ABI values. Vascular calcification associated with specific diseases such as CKD, diabetes mellitus [17], and hyperlipidaemia [18] has received widespread attention, but less attention has been given to the prevalence and management of vascular calcification in the general population. Meanwhile, although an elevated ABI has great significance in predicting vascular calcification [9], its clinical significance has been largely overlooked. Therefore, the prevalence and risk factors for vascular calcification based on ABI values [9] have not been fully assessed and need further exploration in the general population [19, 20].

Based on the above, the purpose of the present study was to investigate ABI values, evaluate the prevalence of vascular calcification according to ABI values, and analyse the risk factors for vascular calcification in the general population. Through this study, the prevalence of vascular calcification was estimated in the general population, and potential methods suitable for implementing general population-based interventions were explored. It is anticipated that our study may shed light on effective preventive measures to eliminate potential risk factors for vascular calcification based on ABI values; thus, improving vascular function and outcomes in the general population.

## Methods

### Research subjects

Our study was a cross-sectional study that enrolled subjects aged 30–70 from the physical examination centre of our hospital from December 2018 to October 2021. Individuals suffering from infectious diseases, trauma, or surgery, or those with a history of organ transplantation, tumours, diabetes, or cardiovascular diseases (including heart failure, coronary artery disease, myocardial infarction, cardiomyopathy, cardiac arrhythmia, and valvular disease), cerebrovascular diseases (including ischaemic stroke and cerebrovascular haemorrhage), liver diseases (including hepatitis, liver cirrhosis, and any abnormal liver function), any kind of kidney diseases, or hyperparathyroidism were excluded from the study.

### Data collection

Clinical and medical history data, such as age, body mass index (BMI) defined as weight/height^2^, systolic blood pressure (SBP), and diastolic blood pressure (DBP), were collected from subjects. Venous blood was drawn from all subjects after a fasting period of at least 8 h. Laboratory indicators such as total cholesterol (TC), triglyceride (TG), high-density lipoprotein cholesterol (HDL-C), low-density lipoprotein cholesterol (LDL-C), serum uric acid (UA), and estimated glomerular filtration rate (eGFR) were tested with fasting blood samples using automatic biochemical analysers in the clinical laboratory of our hospital. An SBP ≥ 140 mm of mercury (mmHg) was defined as elevated SBP, and a DBP ≥ 90 mmHg was defined as elevated DBP according to the consensus of the European Society of Cardiology (ESC)/European Society of Hypertension (ESH) blood pressure guidelines [21]. Fatty liver disease was diagnosed with a colour Doppler ultrasound diagnostic scanner (C1-6 abdominal probe, frequency range 1–6 MHz, LOGIQ E9, American GE) by a trained, experienced sonographer. Lifestyle data including smoking status, alcohol consumption, physical exercise, and postmenopausal status were acquired by questionnaires. Smoking behaviours were divided into never smokers, ex-smokers, and current smokers [22]. The total amount of alcohol consumption was calculated in grams per week and categorized into never drink, minimal alcohol consumption (< 40 g/week), light alcohol consumption (40–140 g/week), moderate alcohol consumption (140–280 g/week), and excess alcohol consumption (> 280 g/week) [23, 24]. Subjects who exercised at least once per week were defined as exercisers, and those who exercised less than once per week were classified as non-exercisers [25]. The postmenopausal status of women was defined as beginning 1 year after the cessation of menses [26].

An automatic arteriosclerosis detector (AS-1000, United Kingdom) was used to measure the brachial artery and the ankle arterial pulsation and systolic pressure of subjects on the left and right limbs in the supine position [27] according to the standard operating procedure of the instrument. The average value of three independent measurements was recorded [27]. The ABI value was calculated as the ratio of average ankle systolic blood pressure divided by the average brachial systolic blood pressure [7]. Those with an ABI value > 0.9 were selected for further analysis. Those with an ABI value ≥ 1.3 were defined as having a high ABI (ABI (+)), which indicates arteriosclerosis, especially vascular calcification [11, 28], and those with an ABI value < 1.3 and > 0.9 were defined as having a normal ABI (ABI (−)) [11, 28].

### Statistical analysis

The SPSS statistical software package, version 24.0, was used for data analysis. The ABI was used as a dichotomous outcome variable (ABI (+) group and ABI (−) group) for data analysis. Continuous variables with normal distribution were presented as the mean ± standard deviation ($\overline{\user2{x}}$** ± s**), and the independent Student’s t-test was performed to compare differences between the two groups. Categorical variables were expressed as numbers (n) and percentages (%), and the Chi-square (χ^2^) test or Fisher’s exact test was used to determine differences between groups. The Kruskal–Wallis test was used to compare ranked data between different groups. All P values were two-sided, and P values ≤ 0.05 were considered statistically significant.

The prevalence of vascular calcification between males and females was compared, and baseline data for the ABI (−) and ABI (+) groups of males and females were also compared. Pearson correlation coefficient analysis was used to identify multicollinearity, and a correlation coefficient of more than 0.9 was considered to represent multicollinearity [29]. Variables with P ≤ 0.05 in univariate analysis or indicators clinically believed or confirmed in previous studies [30] to be associated with vascular calcification and with no multicollinearity of these variables [31] were included in the multivariate logistic regression analysis [32]. The multivariate logistic regression model was constructed using the enter method to screen for the risk factors for vascular calcification, and the odds ratio (OR) and 95% confidence interval (CI) were calculated.

## Results

### Prevalence of vascular calcification based on the ABI in males and females

**Fig. 1:**
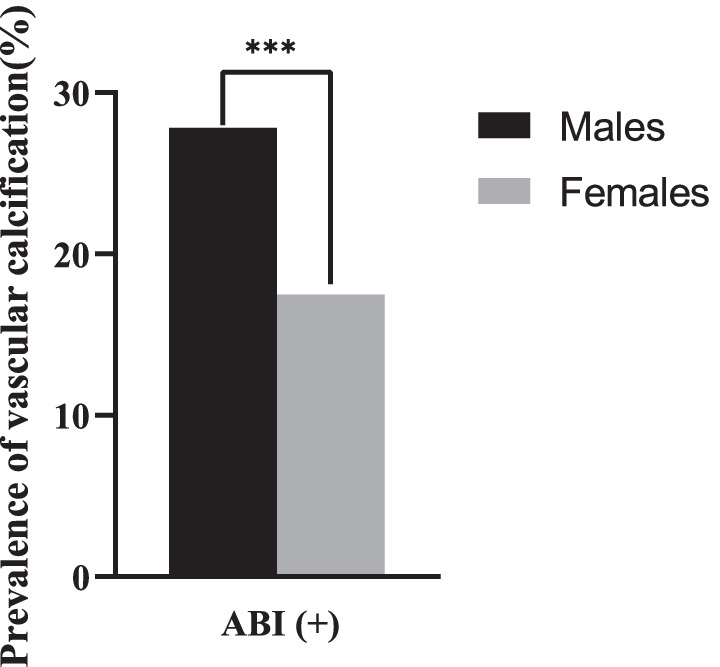
The prevalence of vascular calcification based on the ABI value in males and females

A total of 1033 subjects were enrolled in the study, including 690 males (66.80%) and 343 females (33.20%). The overall prevalence of ABI (+), which indicates vascular calcification, was 24.39% among the 1033 subjects. Among the 690 male subjects, the prevalence of vascular calcification was 27.80% (present in 192 male subjects), while the prevalence of vascular calcification was 17.49% among the 343 female subjects. The prevalence of vascular calcification in males was significantly higher than that in females (*P* < 0.001, Fig. 1).

### Comparison of baseline characteristics between the ABI (−) and ABI (+) groups for males and females

Since the prevalence of vascular calcification in males and females was statistically significant, men and women were divided into two groups for the next analysis. Baseline characteristics were compared between the ABI (+) and ABI (−)groups. The differences in age, BMI, SBP, DBP, TG, HDL-C, hypertension, and fatty liver disease were statistically significant in males (*P* < 0.05); however, no differences were found in UA, TC, LDL-C, serum calcium, serum phosphorus, eGFR, or lifestyle behaviours such as smoking, alcohol consumption, and exercise between the ABI (−) and ABI (+) groups (Table 1). The differences in UA, TC, TG, LDL-C, eGFR, alcohol consumption, exercise, and postmenopausal status were statistically significant in females (*P* < 0.05); however, no differences were found in age, BMI, SBP, DBP, HDL-C, serum calcium, serum phosphorus, smoking, or fatty liver disease between the ABI (−) and ABI (+) groups (Table 1).

**Table 1:** Comparison of baseline characteristics between the ABI (−) and ABI (+) groups for males and females

Variables	Males (n = 690)				Females (n = 343)			
	ABI (−)	ABI (+)	t/**χ**^**2**^/Z value	P value	ABI (−)	ABI (+)	t/**χ**^**2**^/Z value	P value
Age (year,$\overline{x}$ ± s)	50.07 ± 9.30	51.63 ± 8.92	-1.987	0.047	51.72 ± 8.28	50.40 ± 8.52	1.120	0.264
BMI (kg/m^2^, $\overline{x}$ ± s)	23.50 ± 3.04	25.16 ± 2.81	-6.533	< 0.001	22.02 ± 2.92	22.76 ± 3.23	-1.641	0.105
SBP (mmHg, $\overline{x}$ ± s)	122.56 ± 14.44	126.32 ± 14.00	-3.097	0.002	115.12 ± 14.84	113.80 ± 12.57	0.648	0.518
DBP (mmHg, $\overline{x}$ ± s)	78.47 ± 9.24	81.24 ± 9.75	-3.474	0.001	71.34 ± 9.36	72.10 ± 9.32	-0.574	0.567
Hypertension			7.323	0.007			4.264	0.039
No (n (%))	418(83.94)	144(75.00)			260(91.87)	56(93.33)		
Yes (n (%))	80(16.06)	48(25.00)			23(8.13)	4(6.67)		
UA (mg/dl, $\overline{x}$ ± s)	5.84 ± 1.18	5.78 ± 1.09	0.655	0.513	4.11 ± 0.94	4.44 ± 1.13	-2.428	0.016
TC (mmol/l, $\overline{x}$ ± s)	5.35 ± 0.90	5.31 ± 0.86	0.502	0.616	5.55 ± 0.98	4.78 ± 0.63	7.684	< 0.001
TG (mmol/l, $\overline{x}$ ± s)	1.28 ± 0.92	1.47 ± 0.96	-2.408	0.016	0.79 ± 0.05	0.56 ± 0.03	3.231	0.001
HDL-C (mmol/l, $\overline{x}$ ± s)	1.28 ± 0.33	1.20 ± 0.28	2.901	0.004	1.60 ± 0.38	1.66 ± 0.28	-1.253	0.213
LDL-C (mmol/l, $\overline{x}$ ± s)	3.30 ± 0.81	3.29 ± 0.84	0.128	0.898	2.60 ± 0.46	3.32 ± 0.84	-9.318	< 0.001
SC (mmol/l, $\overline{x}$ ± s)	2.16 ± 0.15	2.17 ± 0.22	-0.248	0.805	2.09 ± 0.25	2.04 ± 0.23	0.699	0.487
SP (mmol/l, $\overline{x}$ ± s)	1.16 ± 0.12	1.12 ± 0.19	0.965	0.339	1.29 ± 0.17	1.24 ± 0.12	1.169	0.247
eGFR (ml/min/1.73m^2^, $\overline{x}$ ± s)	90.47 ± 11.70	90.37 ± 9.64	0.112	0.688	92.99 ± 12.51	87.28 ± 10.92	3.280	0.001
Smoking			-1.829	0.067			1.425	0.154
Never (n (%))	148(29.72)	72(37.50)			249(87.99)	45(75.00)		
Previous (n (%))	193(38.75)	68(35.42)			19(6.71)	12(20.00)		
Current (n (%))	157(31.53)	52(27.08)			15(5.30)	3(5.00)		
Alcohol consumption			-0.214	0.830			3.207	0.001
Never (n (%))	108(21.69)	48(25.00)			145(51.24)	18(30.00)		
Minimal (n (%))	148(29.72)	52(27.08)			103(36.40)	12(20.00)		
Light (n (%))	107(21.48)	40(20.83)			25(8.83)	12(20.00)		
Moderate (n (%))	71(14.26)	20(10.42)			7(2.47)	12(20.00)		
Excess (n (%))	64(12.85)	32(16.67)			3(1.06)	6(10.00)		
*Exercise*			1.414	0.234			10.353	0.001
Yes (n (%))	94(18.87)	44(22.92)			225(79.51)	24(40.00)		
No (n (%))	404(81.13)	148(77.08)			58(20.49)	36(60.00)		
*Fatty liver disease*			4.172	0.041			1.324	0.250
Yes (n (%))	322(64.66)	108(56.25)			243(85.87)	48(80.00)		
No (n (%))	176(35.34)	84(43.75)			40(14.13)	12(20.00)		
*Postmenopausal*							4.540	0.033
Yes (n (%))					127(44.88)	36(60.00)		
No (n (%))					156(55.12)	24(40.00)		

ABI: Ankle-brachial index, $\overline{x}$ ± s: mean ± standard deviation, BMI: Body Mass Index, SBP: Systolic Blood Pressure, DBP: Diastolic Blood Pressure, UA: Uric Acid, TC: Total Cholesterol, TG: Triglyceride, HDL-C: High-density lipoprotein cholesterol, LDL-C: Low-density lipoprotein cholesterol, SC: Serum calcium, SP: Serum phosphorus, eGFR: Estimated glomerular filtration rate.

### Multicollinearity analysis of the variables associated with vascular calcification

**Fig. 2:**
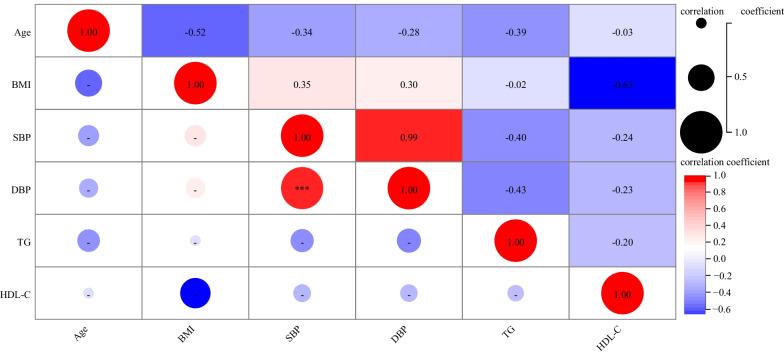
Correlation coefficient matrix of variables in males

**Fig. 3:**
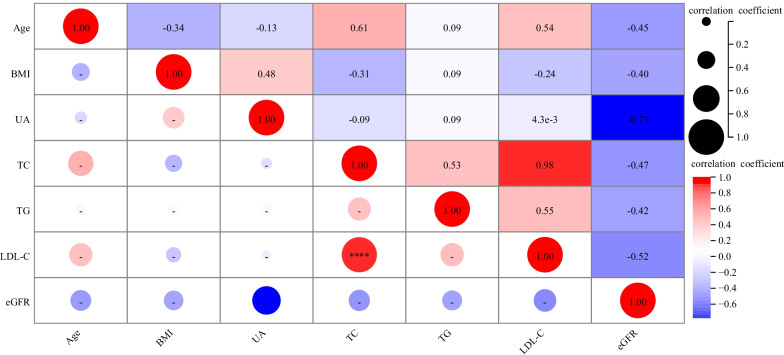
Correlation coefficient matrix of variables in females

After univariate analysis, variables with P ≤ 0.05 and clinically believed or confirmed in previous studies to be associated with vascular calcification were selected for multicollinearity analysis. Age, BMI, SBP, DBP, TG, HDL-C, and fatty liver disease in men were selected for multicollinearity analysis using Pearson correlation analysis. The correlation coefficient between SBP and DBP in the correlation coefficient matrix was significant (r = 0.99, *P* < 0.001, Fig. 2). To avoid multicollinearity, blood pressure was classified into normal blood pressure, elevated DBP, elevated SBP, and elevated SBP and DBP. Then, these classifications were used for multivariate logistic regression analysis. The variables age, BMI, UA, TC, TG, LDL-C, and eGFR in women were selected for multicollinearity analysis, and the correlation coefficient between TC and LDL-C in the correlation coefficient matrix was significant (r = 0.98, *P* < 0.001, Fig. 3). To avoid multicollinearity, the indicator TC was excluded from the multivariate logistic regression model because TC includes HDL-C and LDL-C. However, HDL-C and LDL-C have opposite effects on cardiovascular systems. Therefore, LDL-C was selected for multivariate analysis.

### Multivariate logistic regression analysis of vascular calcification in males and females

**Fig. 4:**
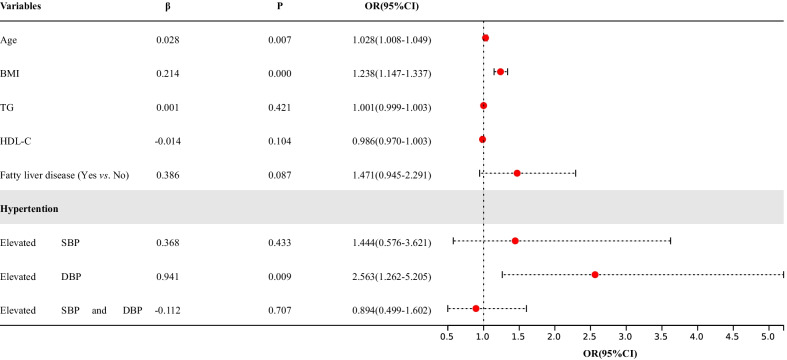
Multivariate logistic regression analysis of vascular calcification in males

After multicollinearity analysis, variables with no multicollinearity were included in the multivariate logistic regression analysis. The results in males showed that increased age (OR = 1.028), increased BMI (OR = 1.238), and elevated DBP (OR = 2.563) were independent risk factors for vascular calcification after adjusting for confounding factors such as TG, HDL-C, and fatty liver disease. The results indicated that each 1.0-year increase in age was associated with a 1.028-fold increased risk of vascular calcification (95% CI 1.008–1.049, *P* = 0.007), each 1.0 kg/m^2^ increase in BMI was associated with a 1.238-fold increased risk of vascular calcification (95% CI 1.147–1.337, *P* < 0.001), and the risk of vascular calcification increased by 2.563-fold in the population with elevated DBP compared with those with normal blood pressure (95% CI 1.262–5.205, *P* = 0.009, Fig. 4).

**Fig. 5:**
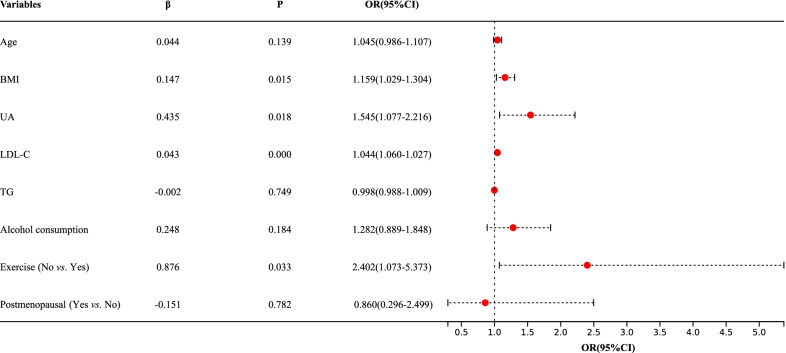
Multivariate logistic regression analysis of vascular calcification in females

The results of multivariate analysis in females showed that increased BMI (OR = 1.159), elevated UA (OR = 1.545), elevated LDL-C (OR = 1.044), and a lack of exercise (OR = 2.402) were independent risk factors for vascular calcification after adjusting for confounding factors such as age, TG, postmenopausal status, blood pressure, and alcohol consumption. The results indicated that each 1.0 kg/m^2^ increase in BMI was associated with a 1.159-fold increased risk of vascular calcification (95% CI 1.029–1.304, *P* = 0.015), each 1.0 mg/dl increase in serum UA level was associated with a 1.545-fold increased risk of vascular calcification (95% CI 1.077–2.216, *P* = 0.018), each 1.0 mmol/l increase in LDL-C level was associated with a 1.044-fold increased risk of vascular calcification (95% CI 1.060–1.027, *P* < 0.001), and women who lacked exercise had a 2.402-fold increased risk of vascular calcification than women who exercised regularly (95% CI 1.073–5.373, *P* = 0.033, Fig. 5).

### Evaluation of the multivariate logistic regression model

To validate and evaluate the accuracy of the multivariate model for males, the Omnibus comprehensive test was performed to comprehensively test the model coefficient, and the P value was 0.000 (*P* < 0.05), indicating that the overall fit of the model was meaningful and quite good. The P value of the Hosmer–Lemeshow goodness-of-fit test was 0.171 (*P* > 0.05), and the prediction accuracy of the fitted model was 72.9% in males (Table 2). There was a good fit for the multivariate logistic regression model in males. The accuracy test was also performed on the multivariate model for females. The P value of the Omnibus test was 0.000 (*P* < 0.05), the P value of the Hosmer–Lemeshow test was 0.347 (*P* > 0.05), and the prediction accuracy was 82.8% in females. The multivariate logistic regression model for females also showed a good model fit.

**Table 2:** The comprehensive evaluation of the fitted model

Comprehensive evaluation indicators	Males	Females
Omnibus test of model	0.000	0.000
Hosmer–Lemeshow test	0.171	0.347
Prediction accuracy (%)	72.90	82.80

## Discussion

Vascular calcification plays an important role in the pathogenesis of CVD because it may progressively lead to many complications, such as increased cardiac afterload, ventricular hypertrophy, reduced coronary artery perfusion, myocardial ischaemia, and cardiac diastolic dysfunction or even heart failure [4], caused by arterial stiffening [33]. Meanwhile, it is also related to a higher risk of dementia, cognitive decline [34], and all-cause mortality [35]. At the same time, vascular calcification is widely accepted as one of the common and important complications of CKD patients who suffer from hyperphosphatemia and accumulation of uraemic toxins [36]. Currently, much attention has been given to the mechanisms and risk factors for vascular calcification in CKD patients [37]. However, the prevalence and related risk factors for vascular calcification have not received much attention in the general population. In our current study, we evaluated the prevalence of vascular calcification according to the ABI value and analysed the risk factors for vascular calcification in the general population. We found that the prevalence of vascular calcification was also high in the general population of our centre. The overall prevalence of ABI (+), which indicates vascular calcification, was 24.39% among 1033 subjects with a mean age of 50.85 ± 8.95 in our survey. The prevalence of vascular calcification based on the ABI value was much higher in men (27.80%) than women (17.49%) on the condition that there was no significant difference in the age of the selected samples (50.51 ± 9.21 years old in males and 51.64 ± 8.30 years old in females, *P* = 0.056). Other previous studies also proved that 61% of asymptomatic subjects displayed vascular calcification [14], and systemic vascular calcification may affect as many as 30%–50% of asymptomatic individuals in the United States [38]. Hence, it is essential to focus on the detection of vascular calcification in the general population so that early prevention and control measures can be carried out promptly.

For the reason mentioned above, we conducted single-factor analyses and multivariate logistic regression analyses to look for risk factors related to vascular calcification in the general population. We found that increased age, increased BMI, and elevated DBP were independent risk factors for vascular calcification in males. We also found that increased BMI, elevated UA, elevated LDL-C, and a lack of exercise were independent risk factors for vascular calcification in females after adjusting for other confounding factors. It is well known that age is a major driver of cardiovascular risk [1], and the aging process of arteries is naturally characterized by a gradual increase in the stiffness and calcification of the vasculature [16]. Although age-driven risk factors cannot be reduced, other controlled factors can be managed to improve vascular function.

For males, elevated DBP and SBP were risk factors for vascular calcification in univariate analysis, while elevated DBP was an independent risk factor for vascular calcification in multivariate logistic regression analysis. Therefore, our study showed a relatively clear correlation between DBP and vascular calcification. However, we must know that hypertension is a manifestation of the decline of vascular elasticity [39] and impaired arterial compliance on account of increased peripheral vascular resistance induced by vascular calcification. Meanwhile, elevated blood pressure can also lead to haemodynamic changes [40] and shear stress-induced injury of vascular endothelial cells and vascular smooth muscle cells [41]. All of these factors contribute to the noncompliance of the arterial wall and deterioration of the process of vascular calcification. However, if severe arterial stiffness exists in both large and small arteries, especially aortic stiffness, there may be a decrease in DBP because of the seriously weakened buffering effect of the hardened vascular wall. Therefore, pulse pressure may also play an important role in assessing vascular function [42] for those populations who have a decreased DBP. It is also known that hypertension and vascular calcification can influence each other and aggravate the progression of each other. A cohort study proved that a high ABI value is associated with higher mortality in patients with hypertension [11], which indicates that the ABI can provide prognostic information for the hypertensive population. In our study, we found no effects of smoking, alcohol consumption, or exercise on vascular calcification in either univariate or multivariate analysis. Many previous studies have confirmed the viewpoint that there is a strong association between cigarette exposure and endothelial dysfunction [43–45]. Thus, we suggest that discontinuing the habit of smoking would also help decelerate the development of vascular calcification and prevent major cardiovascular events. However, there is a complex association between alcohol consumption and cardiovascular disease. Some studies hold the opinion that low-moderate alcohol consumption may be a protective factor for the cardiovascular system, but chronic abuse or binge drinking will aggravate CVD [46], while other studies argue that there is a dose-dependent harmful relationship between alcohol consumption and arterial stiffness [47]. Although there was no statistically significant difference in drinking behaviours between the ABI (+) and ABI (−) groups, the prevalence of ABI (+) in the never, minimal, light, moderate, and excess alcohol consumption groups showed that the prevalence of vascular calcification was much higher in those who consumed alcohol in excess (33.33%). Furthermore, the prevalence of vascular calcification in the moderate alcohol consumption group was much lower (21.99%) than that in those who never consumed alcohol (30.77%), the minimal alcohol consumption group (26.00%), and the light alcohol consumption group (27.21%). For this reason, we deeply believe that excessive alcohol consumption is a risk factor for vascular calcification. However, whether minimal-to-moderate alcohol consumption can protect vascular function is yet to be determined. Therefore, further multicentre and prospective large-sample studies are needed to clarify the dose-dependent association between alcohol consumption and vascular calcification in the general population.

The 2016 ESC guideline revealed that the burden of cardiovascular disease in women is ten years later than that in men [1]. Our study not only confirmed that the prevalence of vascular calcification in men (27.80%) was much higher than that in women (17.49%), but also proved that there was a difference in the prevalence of vascular calcification between postmenopausal women and premenopausal women in univariate analysis. The results showed that the prevalence of vascular calcification in postmenopausal women was much higher than that in premenopausal women (22.09% *vs.* 13.33%), which further proved the protective effect of oestrogen on the cardiovascular system in females. Thus, males appear to develop CVD earlier than females. However, postmenopausal women are faced with a rapidly increased CVD risk, which can also cause adverse cardiovascular events. A previous systemic evaluation of calcified atherosclerosis that enrolled 650 asymptomatic subjects revealed that 47% of women younger than 50 years old were found to have arterial calcification, but the prevalence rapidly increased to 73% between the ages of 50 and 60 and further increased to 91% between the ages of 60 and 70 [38]. This study also showed that 70% of men younger than 50 years old were found to have arterial calcification, and the prevalence increased to 92% between the ages of 50 and 60 and further increased to 98% between the ages of 60 and 70 [38]. All of these results further demonstrate that vascular calcification may develop at an accelerated rate in females after menopause. In multivariable analysis, the postmenopausal status was not an independent risk factor for vascular calcification in women, which may be partly because oestrogen reduction is also related to obesity. Hence, the increased BMI may mask the effects of menopause. This is consistent with the phenomenon that females with polycystic ovary syndrome (mainly combined with decreased oestrogen and obesity) usually have a higher risk of CVD [41].

For females, our results also showed that there was a statistically significant difference in UA between the ABI (−) and ABI (+) groups in the univariate comparison. The mean level of serum UA in the ABI (+) group was much higher than that in the ABI (−) group, and this significant association persisted in the multivariate analysis after adjusting for potential confounders. Some studies proposed that asymptomatic hyperuricaemia was independently associated with the severity of coronary calcification [48, 49] and that the serum level of UA showed a positive correlation with the abdominal aortic calcification score in a dose–response manner [50, 51]. In our current study, increased UA was also an independent risk factor for vascular calcification for females, and each 1.0 mg/dl increase in serum UA was associated with a 1.545-fold increased risk of vascular calcification. Therefore, we speculate that the increased level of serum UA plays an important role in vascular calcification in females. UA is an end product of purine catabolic metabolism. The serum level of UA may be influenced by exogenous ingestion, endogenous production by the liver, and renal excretion. However, the serum level of UA is determined mainly by dietary intake (high purine diet) in the general population [52]. Therefore, clinical indicators are closely related to lifestyle habits and diet in the general population. It has been proved that the Mediterranean diet may reduce the risk of CVD by 29% over a 5-year period [53]. ESC guidelines indicate that all people < 50 years old with elevated CVD risk factors should pay attention to lifestyle behaviours (with emphasis on avoiding smoking, being overweight and sedentary behaviour) to reduce CVD risk factors [40]. Our results also proved that physical inactivity was an independent risk factor for vascular calcification and that dietary habits played a pivotal role in vascular calcification because elevated UA (high purine) and LDL-C (high-fat diet) were all independent risk factors for vascular calcification. Meanwhile, BMI was an independent risk factor for vascular calcification in both men and women in the current study. This result is consistent with research performed in the general population of South China showing that BMI is an effective predictor for high ABI values [28]. Therefore, we can conclude that lifestyle intervention may play a pivotal role in controlling the progression of vascular calcification.

Since our study was a survey of the general population, there was no difference in calcium and phosphorus levels between the vascular calcification group and the nonvascular calcification group in both men and women. However, patients with certain diseases, such as CKD patients suffering from hyperphosphatemia, diabetes mellitus [17], or hyperlipidaemia [18], may carry a greater risk for developing vascular calcification than the general population. In the current study, we analysed the serum calcium, serum phosphorus, TG, TC, renal function, and history of diabetes, but we did not assess the exact levels of blood glucose, which may be associated with the ABI value [54]. Therefore, additional studies are required to further investigate the relationship between blood glucose and vascular calcification. Furthermore, the results of this study are only applicable to the general population. Further studies are needed to identify predictors for the target population with a certain disease.

A high ABI value is a marker of subclinical vascular conditions, including atherosclerosis, vessel stiffness, and calcification [55]. Therefore, a high ABI value can be used as a method for screening vascular calcification with the advantages of being highly portable, inexpensive, and requiring no radiation. However, it cannot distinguish the specific vascular calcification bed. Doppler ultrasound can be used to determine the location of the calcified vessels, but it can only be applied to superficial vessels. Although plain X-ray, computed tomography, or magnetic resonance imaging can provide the location of the calcified vessel beds, these methods may expose the individuals to substantial radiation. Therefore, we suggest the use of the ABI as a method to screen for vascular calcification and identify individuals who are at high risk of vascular calcification. Then, Doppler ultrasonography, planar radiographs, computed tomography or magnetic resonance imaging can be performed to provide more detailed information about calcified vascular beds for high-risk individuals. Meanwhile, targeted preventive interventions can be carried out for high-risk individuals according to the results of risk factor analysis to delay the progression of vascular calcification.

In summary, risk assessment of vascular calcification based on the ABI is of great significance for the early diagnosis and treatment of vascular calcification-related CVD events. Targeted preventive interventions for vascular calcification should also be considered in the general population. The impending clinical and economic burden of high-risk populations can be curbed by promoting healthy lifestyle behaviours and addressing clinical risk factors.

## Conclusions

The prevalence of vascular calcification based on the ABI is also high in the general population. The prevalence of vascular calcification in males is much higher than that in females, and the risk factors for vascular calcification are different in males and females. Increased age, BMI, and elevated DBP are independent risk factors for vascular calcification in males. Increased BMI, UA, LDL-C, and a lack of exercise are independent risk factors for vascular calcification in females. Attention should be given to strengthening the prevention and control of vascular calcification in the general population.

## Limitations

The present study exhibits some limitations. Our study was a single-centre cross-sectional study that can only provide correlation relationships rather than causality relationships. Some unmeasured or unknown factors may also be associated with the ABI value. Thus, multicentre prospective cohort studies with more comprehensive indicators are needed to further verify our results and clarify the cause-and-effect relationships.

## Data Availability

The data analysed during the current study are not publicly available because there are many other kinds of information in our recruited dataset; however, the data are available from the corresponding author upon reasonable request.

## Abbreviations

ABI: Ankle-brachial index
BMI: Body mass index
SBP: Systolic blood pressure
DBP: Diastolic blood pressure
TG: Triglyceride
HDL-C: High-density lipoprotein cholesterol
UA: Uric acid
TC: Total cholesterol
LDL-C: Low-density lipoprotein cholesterol
eGFR: Estimated glomerular filtration rate
OR: Odds ratio
CI: Confidence interval
CVD: Cardiovascular disease
CKD: Chronic kidney disease
PAD: Peripheral artery disease
mmHg: Millimetres of mercury
ESC: European Society of Cardiology
ESH: European Society of Hypertension
